# Building Compassionate Experience Through Compassionate Action: Qualitative Behavioral Analysis

**DOI:** 10.2196/43981

**Published:** 2023-05-31

**Authors:** Laura Desveaux, Kelly Wu, Geneviève Rouleau, Diya Srinivasan, Rhea Azavedo, Marlena Dang Nguyen, Danielle Martin, Carolyn Steele Gray

**Affiliations:** 1 Institute for Better Health Trillium Health Partners Mississauga, ON Canada; 2 Institute for Health System Solutions and Virtual Care Women's College Hospital Toronto, ON Canada; 3 Institute for Health Policy, Management & Evaluation University of Toronto Toronto, ON Canada; 4 Department of Family and Community Medicine University of Toronto Toronto, ON Canada; 5 Lunenfeld-Tanenbaum Research Institute Sinai Health System Toronto, ON Canada

**Keywords:** technology-based care, compassion, primary care, behavior change, communication competency, continuing professional development, qualitative

## Abstract

**Background:**

The acceleration of technology-based primary care during the COVID-19 pandemic outpaced the ability to understand whether and how it impacts care delivery and outcomes. As technology-based care continues to evolve, focusing on the core construct of compassion in a primary care context will help ensure high-quality patient care and increased patient autonomy and satisfaction. The ability to successfully operationalize the use of technology in patient-clinician interactions hinges on understanding not only *how* compassionate care is experienced in this context but also how clinicians can create it.

**Objective:**

The objectives of this study were to understand whether and how compassionate behaviors are experienced in technology-based primary care interactions and identify the individual and contextual drivers that influence whether and how these behaviors occur.

**Methods:**

We conducted a series of qualitative one-on-one interviews with primary care physicians, nurses, and patients. Qualitative data were initially analyzed using an inductive thematic analysis approach to identify preliminary themes for each participant group independently. We then looked across participant groups to identify areas of alignment and distinction. Descriptions of key behaviors that participants identified as elements of a compassionate interaction and descriptions of key drivers of these behaviors were inductively coded and defined at this stage.

**Results:**

A total of 74 interviews were conducted with 40 patients, 20 nurses, and 14 primary care physicians. Key behaviors that amplified the experience of compassion included asking the patient’s modality preference, using video to establish technology-based presence, sharing the screen, and practicing effective communication. Participants’ knowledge or skills as well as their beliefs and emotions influenced whether or not these behaviors occurred. Contextual elements beyond participants’ control influenced technology-based interactions, including resource access, funding structures, culture, regulatory standards, work structure, societal influence, and patient characteristics and needs. A high-yield, evidence-based approach to address the identified drivers of compassion-focused clinician behavior includes a combination of education, training, and enablement.

**Conclusions:**

Much of the patient experience is influenced by clinician behavior; however, clinicians need a supportive system and adequate supports to evolve new ways of working to create the experience of compassionate care. The current state of technology-based care operationalization has led to widespread burnout, societal pressure, and shifting expectations of both clinicians and the health system more broadly, threatening the ability to deliver compassionate care. For clinicians to exhibit compassionate behaviors, they need more than just adequate supports; they also need to receive compassion from and experience the humanity of their patients.

## Introduction

### Background

The novel COVID-19 pandemic prompted a rapid shift to technology-based care in health systems around the world. In Ontario, Canada, there was a 79.1% reduction in office-based primary care visits and a 56-fold increase in technology-based visits [1], defined as any synchronous visit conducted via either telephone or video call. This shift was similar in outpatient ambulatory care: the proportion of Ontario residents who had a technology-based visit increased rapidly to 29.2% in the second quarter of 2020, with most visits (91.2%) being conducted via phone [2]. A similar trend was observed in the National Health Service, with reports of technology-based visits comprising 50% to 90% of outpatient activities [3,4]. Although the acceleration of technology-based care may have both benefits and drawbacks [5,6], this rapid uptake outpaced the ability to understand whether and how it impacts care delivery and outcomes.

Although the benefits of greater convenience and flexibility should be celebrated, connectivity is central to the ability to recognize and respond to the suffering of others. It is a key element of compassionate care [7], which is defined as an *awareness* of suffering and a subsequent *action* to relieve it [8]. Compassion is a widely reflected health system value that supports the sustainability of high-quality patient care [9] and increased patient autonomy and satisfaction [10]. It is also an explicit core value in primary care and family medicine [11], where longitudinal relationships built on compassionate care are the key to clinical effectiveness. Although technology has supported elements of health communication, such as knowledge exchange, education, and decision support [12], the technology-based and in-person experiences of or approaches to compassion may not be the same [13]. As technology-based care continues to evolve, the ability to successfully operationalize the use of technology in patient-clinician interactions hinges on understanding *how* compassionate care is experienced in this context [14].

### Goal of This Study

There is a need to couple our understanding of the patient experience of compassion with the identification of the “range of concrete techniques that physicians may engage in that are experienced as compassionate by patients” [15]. Simply put, it is not enough to understand the ideal experience of care; how clinicians can create it must also be understood. Early work has described categories of action, including listening and paying attention, following up and running tests, and providing holistic care [15]; however, these categories do not specify concrete actions. Furthermore, the actions and behaviors characterizing in-person interactions do not directly translate to technology-based encounters [16], necessitating the need for context-specific studies. There is an opportunity to close the gap patients experience between in-person and technology-based encounters by establishing a shared understanding of the specific behaviors that characterize a technology-based encounter and how these can foster a compassionate experience. Therefore, the objectives of this study were to (1) understand whether and how compassionate behaviors are experienced in technology-based primary care interactions and (2) identify the individual and contextual drivers that influence whether and how these behaviors occur.

## Methods

### Study Design

This qualitative study involved one-on-one, semistructured interviews conducted with patients and primary care clinicians, including family physicians and nurses (including nurse practitioners and registered nurses). The interview questions explored how technology influences interactions in primary care, with a focus on understanding how compassionate experiences can be supported for both patients and clinicians. For the purposes of this study, technology-based care was defined as any health care interaction that occurred via technology, including video visits, asynchronous messaging (eg, email), and remote monitoring. The reporting of this study was guided by the COREQ (Consolidated Criteria for Reporting Qualitative Research) checklist [17].

### Study Setting

The Canadian health care system provides public funding for universal hospital and physician services to all eligible residents, making it free at the point of care for these services [18]. Family physicians and nurses funded under this model provide primary care services to patients, including health promotion, preventative care, and the management of acute and chronic conditions [19]. Primary care clinicians are considered patients’ first point of contact to the health care system, where care is designed to be relationship based and longitudinal [20]. Whether and how patients can access technology-based care is at the discretion of the clinician; means of technology-based care may include but is not limited to technology-based video visits and asynchronous messaging. This access increased substantially after the onset of the COVID-19 pandemic necessitated social distancing, leading to a surge in the use of technology-based care [1], which was supported by quick changes in funding and policies to enable use [21].

### Sampling and Participant Recruitment

Three groups of participants were invited to take part in the study: (1) patients, (2) primary care physicians, and (3) primary care nurse practitioners and registered nurses. Patients were eligible if they lived in Canada, regardless of whether they had experience with technology-based care. Primary care clinicians were eligible if they were actively practicing in Canada and had experience providing technology-based care through at least one of the following means: video visits, asynchronous messaging (eg, email communication and SMS text messaging), and remote monitoring technologies. Given the importance of body language and emotional cues as well as the surge in video visits at the onset of the COVID-19 pandemic [1], telephone visits were not included in the definition of technology-based care; therefore, clinicians were not eligible if their experience of technology-based visits was limited to the use of telephone as a modality.

### Recruitment Strategy

Multipronged convenience sampling was used to recruit patients and clinicians by leveraging social media platforms (ie, Twitter [Twitter, Inc], LinkedIn [LinkedIn Corporation], and Facebook [Meta Platforms, Inc]) and health service organizations (Multimedia Appendix 1). We engaged with a patient partner group, Equity-Mobilizing Partnerships in Community (EMPaCT), during the early stages of the study to receive guidance on our study methods. EMPaCT is a patient-partnership model based at the Women’s College Hospital, an academic health sciences center in downtown Toronto. EMPaCT provided a health equity assessment for the study protocol and advised on best practice recruitment strategies for reaching diverse communities underrepresented in health research.

Interested patients and primary care clinicians reached out to the primary study contact via email, after which their eligibility for the study was determined. A snowball recruitment strategy was then used, wherein included participants were asked to refer colleagues or contacts who may provide relevant insights to the study, including divergent opinions. A follow-up recruitment email was sent within 2 weeks of the original invitation. All eligible participants who expressed interest were sent a study information letter and consent form by email at the time of scheduling the interview, with all participants given a minimum of 48 hours to review the information. A consent checklist was then reviewed before the interview to address any questions the participant had and obtain consent. The researchers had no established relationships with any of the participants before the start of the study and were not involved in their care.

### Data Collection

Individual qualitative semistructured interviews with patients, family physicians, and nurses were conducted by 1 member of the research team (KW, MDN, or GR) between April and November 2021. The interviews lasted between 30 and 45 minutes and were conducted over Zoom (Zoom Video Communications, Inc), a web-based videoconferencing platform. A second team member (RA) was present with their camera and microphone turned off to take notes and observe the interviews to help assess saturation. Recruitment continued until thematic saturation was determined to have been reached, meaning that little to no new comments or insights emerged during the interviews that either refined or challenged the existing insights or categories of insights within the data sample [22].

The interview guides were tailored to each participant group (Multimedia Appendix 2). Interviews with patients and clinicians who had experience with technology-based care included questions aimed at understanding their experiences with compassionate care, perceived challenges and benefits of technology-based care, and perspectives on how to best use technology-based care with respect to compassionate care. Interviews with patients who had no experience with technology-based care explored their experiences with compassionate care and their perceptions and beliefs about technology-based care. The patient interview guide was reviewed by EMPaCT but was not otherwise formally pilot-tested with patients or clinicians. All interviews were audio recorded, anonymized, and transcribed verbatim by a third party. The transcripts were not returned to participants for comment.

### Ethics Approval and Informed Consent

This study was formally reviewed by institutional authorities at the Women’s College Hospital and was deemed not to require research ethics board approval under the Assessment Process for Quality Improvement Projects (APQIP) pathway (APQIP # 2021-0028-P). Participants were informed that participation in the interview was completely voluntary and that they could withdraw from the study at any time without penalty. Verbal informed consent was obtained before the start of the interviews, and participants were given an electronic gift card in recognition of their time. Honorarium rates were CAD $30 for patients, CAD $75 for nurses, and CAD $100 for physicians, with a conversion rate of CAD $1=US $1.30 at the time of the study. Demographic information, including age, gender, and ethnicity, was collected from all participants before the interview. These data were anonymized and stored separately from the transcripts, which were deidentified and stored on a secure server.

### Data Analysis

Qualitative data were initially analyzed using an inductive thematic analysis approach [23,24]. In the inductive phase, the research team deeply engaged with the data by reading the transcripts and documenting any emerging thoughts and reflections. In the second phase, a subset of 3 transcripts chosen at random for each participant group was coded by at least 2 team members (among KW, MDN, GR, RA, and DS) independently. The team members then came together to discuss open codes for the transcripts that they had independently coded. A preliminary coding framework was then created for each participant group. The remaining transcripts were divided among 4 team members (KW, MDN, GR, and DS), who independently single coded using the coding framework. The team met regularly to iteratively refine the coding framework to reflect new codes and merge related codes. NVivo software (versions 11 and 12, QSR International) was used to assist with coding and analysis. Codes were then regrouped into preliminary themes by participant group and were mapped back to the study objectives. Miro (RealtimeBoard, Inc), a web-based whiteboard collaboration tool, was used to aid the research team in visually representing and identifying the relationships between the preliminary themes. Miro boards were also used as a consultation strategy to facilitate reflection on the findings with the broader research team; the team not only reviewed the Miro boards in meetings but could also independently review and comment on the boards. We then began to look more broadly across participant groups to identify areas of alignment and distinction. Descriptions of key behaviors that participants identified as elements of a compassionate interaction and descriptions of key drivers of these behaviors were inductively identified and collated at this stage. As not all behavioral drivers identified by participants were described in the context of a specific behavior, general attributes of context were mapped to the categories identified by Squires et al [25] (eg, financial attributes, system features, work structure, and culture). The ongoing and iterative inductive process involved triangulating data across participant groups and reviewing and defining the themes. We created a thematic summary for each participant group, in which themes and subthemes were named, defined, and supported by key quotes.

A visual representation of the themes was created and reviewed to achieve consensus among all members of the research team (LD, KW, GR, DS, and CSG). An audit trail of all team meetings including audio recordings, meeting minutes, all versions of the coding framework, and thematic summaries was maintained. Finally, a summary of the findings was sent to participants who consented to participate in member checking to ensure the accuracy of our findings.

## Results

### Overview

A total of 74 interviews were conducted with 40 patients, 20 nurses, and 14 primary care physicians, of whom 13 (32%) out of 40 patients, 3 (15%) out of 20 nurses, and 1 (7%) out of 14 physicians participated in member checking. The interviews ranged from 23 to 72 (mean 42) minutes in duration. Participant characteristics are listed in Table 1. The analysis identified clinician behaviors that establish and amplify compassionate care as well as the factors that drive these specific behaviors. The final theme describes a range of contextual factors that influence behavior more generally in practice.

**Table 1:** Demographic characteristics for interview participants.

		Participant group		
		Patient (n=40), n (%)	Nurse (n=20), n (%)	Physician (n=14), n (%)
**Age range (years)**				
	18-20	4 (10)	0 (0)	0 (0)
	21-29	11 (28)	1 (5)	1 (7)
	30-39	10 (25)	13 (65)	5 (36)
	40-49	5 (12)	0 (0)	4 (29)
	50-59	1 (2)	1 (5)	0 (0)
	≥60	6 (15)	1 (5)	3 (21)
	Unidentified	3 (8)	4 (20)	1 (7)
**Gender**				
	Female	26 (65)	20 (100)	9 (64)
	Male	14 (35)	0 (0)	5 (36)
**Ethnicity**				
	Black African	4 (10)	1 (5)	0 (0)
	Black Caribbean	1 (2)	0 (0)	0 (0)
	Black North American	6 (15)	0 (0)	0 (0)
	East Asian	3 (8)	2 (10)	2 (14)
	Latin American	1 (2)	0 (0)	0 (0)
	Middle Eastern	0 (0)	1 (5)	0 (0)
	Mixed heritage	0 (0)	1 (5)	0 (0)
	South Asian	6 (15)	1 (5)	1 (7)
	White European	18 (45)	14 (70)	11 (79)
	Unidentified	1 (2)	0 (0)	0 (0)
**Geography**				
	Central East Ontario	24 (60)	9 (45)	3 (21)
	Central West Ontario	3 (8)	0 (0)	0 (0)
	Eastern Ontario	5 (12)	1 (5)	0 (0)
	Northern Ontario	0 (0)	0 (0)	0 (0)
	Western Ontario	3 (8)	6 (30)	8 (57)
	Quebec	3 (8)	0 (0)	0 (0)
	British Columbia	0 (0)	1 (5)	2 (14)
	Unidentified	2 (5)	3 (15)	1 (7)

### Concrete Clinician Behaviors That Establish and Amplify Compassionate Care Experiences

#### Overview

All participants acknowledged that technology-based care changes the way patients and clinicians interact and experience care and impacts the ability to provide reassurance or establish physical presence, which is often associated with an “in-person” encounter (eg, the compassionate action of using physical touch to reassure). Clinician and patient participants described several behaviors unique to technology-based interactions that supported compassionate care experiences (Textbox 1).

**Behaviors that amplify compassion in a technology-based environment.**

- Asking patient’s modality preference
- Using video to establish technology-based presence
- Sharing the screen
- Practicing effective communication
  - Avoiding jargon
  - Making eye contact
  - Asking open-ended questions
  - Setting expectations
  - Engaging in small talk
  - Understanding and clearing doubts
  - Including caregivers
  - Following up on prior concerns
  - Asking probing questions
  - Acknowledging facial expressions and body language

#### Asking Patient’s Modality Preference

Participants expressed the importance of asking their modality preference before the visit, which they suggested could be facilitated by questioning patients about this at the time of booking or by introducing an intake process. Patient participants described that a comprehensive intake process could gather patients’ digital health preferences to inform the tailoring of the technology-enabled approach to ensure the preferred mode of communication, remote interpretation requirements, the level of caregiver engagement, and accessibility needs, among others, and ensure that they are integrated into decision-making.

#### Using Video to Establish Technology-Based Presence

Participants reported that using video established a visual presence and supported both patients’ and clinicians’ ability to observe and respond to nonverbal cues and facial expressions, which could reinforce their experience of compassion. It also enabled patients to communicate specific information (eg, by demonstrating a movement or pointing to an area) that they would otherwise have trouble expressing or describing to their clinician over the phone. The use of video provides clinicians with a potential source of information that would help signal when a patient was confused or required additional support:

I think it was wonderful, because I could actually see the person who I was speaking to, I come from a very expressive culture, and so we are always reading non-verbal cues. So, it was very useful to me to actually see the other person on that side, because I could tell if they were listening to me, especially because where I come from, when you do mm-hmm, where I come from, it’s like you’re bothering me, quit talking, so. But with the [Ontario Telemedicine Network] it reassured, it reinforces even though I’ve been living here forever I know mm-hmm means I’m following, keep going. But it reinforces that we’re actually on the same page, it worked nicely for me.

Patient 23, female participant

Video also reassured patients that they were the focus of attention, with several patients noting the lack of external distractions they unconsciously observed in the office (eg, no chatter from the waiting room, phone ringing, or knock on the door). Similarly, video provides clinicians with the benefit of observing the patient’s living environment, giving them a more holistic understanding of the patient and an opportunity to identify any elements that would deserve to be investigated and followed up on. Although the benefits of video were shared across participants, some also described the convenience and efficiency they enjoyed via telephone visits for receiving test results or routine care:

[With video] you can actually show that you’re listening because again like you could be on the phone and then you could just be like scrolling on your phone and like they don’t know if you’re actually there, if you’re actually listening or you’re distracted with something else, you’re doing some other paperwork. So I think having that face-to-face is helpful to actually show that you’re there and you’re listening and there’s no other distractions going around.

Nurse 8, female participant

#### Sharing the Screen

Some patients appreciated when clinicians used screen share to share relevant information (eg, test results). The screen share feature became an instrument for facilitating the communication between the clinician and patient:

[My doctor] realizes that I’m an anxious person and I’ve had many serious health issues myself. So, when I ask her about test results, for instance, she’ll give me very clear information, show me the screen of the computer. Which is my health record, but how often does that happen, probably not that often. And lay out the results but then talk about the implications in a very matter-of-fact way. And knowing that that information is important to me, that I want to know the facts, but then she’ll talk about the relative importance or risk and make it clear that when I do need to worry when I don’t need to worry.

Patient 25, female participant

#### Practicing Effective Communication

Both patient and clinician participants cited effective communication skills as a key capability for both parties, which they described as including the following concrete behaviors: *avoiding jargon, making eye contact, asking open-ended questions,* and *setting expectations* (eg, describing what is appropriate for technology-based care and what the patient should expect from a technology-based encounter). Patients described that taking the time to *engage in small talk, include caregivers, understand and clear doubts,* and *follow up on concerns* *identified in prior visits* further amplified compassion. Clinicians can explicitly state that they are interested, concerned, and willing to help to account for the learning curve associated with adjusting to technology-based care modalities. Participants further described the importance of active listening, whereby a clinician would *ask probing questions* and *acknowledge and respond to body language or facial expressions* to demonstrate to the patient that they are seen, heard, respected, and understood. Conducting video visits during the pandemic offered patients and clinicians the added benefit of seeing each other’s facial expressions without being hidden by a mask.

### Understanding the Individual-Level Drivers of Compassionate Behaviors in Technology-Based Care

Participants described how their individual knowledge or skills, as well as beliefs and emotions, influenced the specific behaviors described earlier.

#### Gaps in Knowledge and Skills Undermine Effective Communication Behaviors

Clinicians described a range of challenges in translating effective communication skills to a technology-based platform. They described the need for several specific skills, including developing technological capabilities, adapting their professional role and activities to a technology-based medium, and building technology-based compassionate competence. Participants described experiences with technology-based care as more “formal” and problem centered, which they contrasted with the opportunities for more *small talk* with in-person care. Clinicians described gaps in both knowledge and confidence around which behaviors were most effective in a technology-based encounter to establish similar opportunities to those in an in-person encounter:

I think [active listening is important for virtual care]. I think feeling comfortable in the technology itself is beneficial, because then your focus doesn’t have to be on the tech side, it can actually be on providing that care that you want to, to the patient. And I think, at least initially during COVID, that was a huge focus, was again, going back to me being a beginner nurse, you’re so focused on the tasks, versus being there, and the patient. So, the compassion is there, but it’s not as heavy as if you’re able to autonomously function while seeing a patient on the screen and typing in their chart. When you get to that certain ability where you’re not as stressed about the tech side, I think the compassion would definitely increase, just because you’re able to focus more attention on that purely.

Nurse practitioner 4, female participant

#### Shifting Expectations Have Led to Widespread Burnout, Which Impedes Effective Communication Behaviors

Physician and nurse participants reported higher volumes of patient visits following the rapid uptake of technology-based care (compared with the volumes of patients visits before the pandemic, when technology-based use was limited) and a widespread shift in expectations. Specifically, clinicians experienced increased pressure through 2 mechanisms. The first was perceived disconnect between their actual availability and what they felt patients expected of them—that technology-based visits increased convenience and saved time for patients did not mean that convenience similarly increased for clinicians. The second was an absence of clear guidelines outlining what is appropriate for technology-based visits versus in-person visits for supporting their ability to *set expectations* with patients, leading to misalignment around the perceptions of appropriateness. Clinicians described that this misalignment translated into a perceived loss of respect and value for their time among their patients. This compounded with the context of the primary care environment, including a lack of technological support and best practice guidance, to create widespread experiences of burnout across all clinicians, which impacted their ability to provide compassionate care in a fulsome manner. Some clinicians described being reenergized when patients express gratitude or appreciation for the care they receive and the convenience provided by technology-based care, even against the constant cycle of “catch-up” they were experiencing in their practices. There was also broad recognition among both clinicians and patients that appropriate guidance and a triage model would help *set expectations* and would support the effective management of nonurgent and low-acuity concerns:

I definitely do and I think part of it comes out to like burnout as part of being a nurse. It’s like when you feel like your care is being reciprocated—you feel like somebody is actually—is like really thankful for your care and the time that you’re putting in to helping them and coordinating with trying to get them an answer or get them the help they need. When you feel that like appreciation or you see a patient—if you see a patient’s mood change or like their situation improve because of something that you’ve done, of course it’s easier to want to do it more. Like of course you want to provide that care more, like do that more for them and help them out more. It definitely plays a role I think in providing that compassionate care.

Registered nurse 1, female participant

#### Concerns About the Quality of Care and Patient Safety Influence Motivations for the Use of Video

Although clinicians endorsed a hybrid model of technology-based and in-person care, challenges with effective communication led to concerns that the ubiquitous *use of video* instead of in-person care would reduce the quality of care or patient safety. Difficulties in building rapport with patients and gathering relevant information led both physicians and nurses to worry about the occurrence of missed diagnoses in the absence of an in-person assessment, which they felt could become a professional liability. These fears created hesitancy and skepticism around using technology-based care and highlighted a need for clarity on the appropriateness of technology-based modalities in line with patient needs to enable safe and high-quality care:

I don’t think everybody needs to come in. But I also think that there has to be some rule, like once a year to have to see the patients. I don’t know, some sort of—because otherwise we miss those kids with the eating disorders. I’ve missed a gal who thought she lost a ton of weight because she finally—it clicked. And she’s got terrible cancer, right? So we’re missing stuff when you never see the patients. And we all know that intuitively, but actually having too many of those stories is upsetting so now I make people come in periodically.

Physician 6, female participant

There was a couple of things I was very skeptical about it and nervous about it. I kept thinking oh boy, in a year I’m going to have a lawsuit against me. I’m probably going to misdiagnose something or maybe I won’t take something as seriously as I would if it was in person.

Nurse practitioner 17, female participant

### Contextual Attributes of Primary Care Drive Clinician Behavior in Technology-Based Care Interactions

#### Overview

Physician and nurse participants highlighted elements of the primary care context beyond their control that influenced how they generally behave in a technology-based interaction (ie, drivers that participants mentioned but did not associate with a specific concrete behavior). Multimedia Appendix 3 describes these drivers, including resource access, funding structures, culture, regulatory standards, work structure, societal influence, and patient characteristics and needs (refer to Multimedia Appendix 3 for descriptions and supporting quotes). Although there is conceptual overlap with some of the drivers described earlier in the *Results* section, participants mentioned that these drivers specifically influence “general behaviours,” meaning that their influence extends beyond the concrete behaviors described earlier.

#### Resource Access and Funding Structures

Clinicians reported a lack of centralized support for planning and delivering technology-based care. For example, many clinicians described how technological support would help both clinicians and patients navigate the technology required for video visits. Clinicians also expressed having insufficient time to learn a new way of working and a corresponding lack of infrastructure to support the shift to technology-based care. Patient participants also acknowledged the impact of funding structures and described feeling rushed as a function of being limited to one health concern per visit owing to their perception that payment models are time focused and not patient focused.

#### Culture and Regulatory Standards

The prevailing believes were that building connections through in-person consultation is seen as the core of primary care and that technology-based connections are complementary to this core over time. Physicians and nurses emphasized the importance of practicing as a competent and accountable clinician but expressed challenges in showing up as one in technology-based interactions because of a lack of evidence-based standards and best practice guidance. Clinicians described that maintaining the quality of care and patient safety is of utmost importance, and there were mixed perspectives regarding whether and how quality and safety could be maintained using technology. This tension was most acute in circumstances where there was no clear mechanism for replicating the components of in-person presence required to perform physical assessment or provide reassurance (eg, handing a patient a tissue) in a technology-based format:

But the other half of it is people who are using this to, let’s say...they say, oh, my bladder is bothering me, and they just get a standard prescription for an antibiotic without any investigation, any counselling about what to do. This is inappropriate, it doesn’t even meet the standard of a walk-in clinic where you do a urine dipstick for somebody before you give them a prescription or not. I think that is—it’s not even a missed opportunity, it’s malpractice. But no one’s doing anything about it, and it’s establishing a new standard of care which is going to be worse than what we have now.

Physician 10, male participant

#### Societal Influences and Work Structure

Clinicians highlighted a tension among their focus on and subsequent concerns about the quality of care, a health care system that cites a “digital first” growth strategy [26], and a patient’s focus on convenience. The current hybrid model of care disrupted work structures, creating inefficiencies and unanticipated pressures. For example, some clinicians described planning buffer time when conducting video visits, given the potential technical issues that could be experienced by either party. Unpredictable delays negatively impacted subsequent technology-based and in-person visits:

I always book my videos for longer, because you’re thinking, give a little bit for tech time, or if the person’s not quite ready. And usually they’re a more sensitive issue, or something that just didn’t work on the phone, so let’s do a video, so they’re longer. And probably just that time in and of itself lends to more compassionate care.

Nurse practitioner 5, female participant

#### Patient Characteristics and Needs

All participants acknowledged the role of patient characteristics and needs, specifically previous experience with technology, digital literacy, access to technology, and stable internet connectivity, in supporting or impeding compassionate technology-based care. Patients also described the importance of a quiet and confidential space, which impacts both their security and confidence to communicate effectively and disclose sensitive information, allowing them to feel supported during their care visit.

## Discussion

### Principal Findings

This study highlights that much of how patients experience compassion in a technology-based encounter is a product of clinician behavior. The results describe a range of concrete clinician behaviors that contribute to this experience in the context of primary care and a subset of patient behaviors that influence how clinicians experience technology-based care. Our data emphasize a wide array of factors that influence the enactment of compassionate behaviors, underscoring the need to support clinicians in achieving the objective of compassionate technology-based care. Our findings stress the need for best practice recommendations and point us toward areas of strategic focus for health systems to move further along their journey of integrating technology-based care.

### Supporting the Implementation of Compassionate Technology-Based Care

As health systems progress toward their goal of digital transformation, there are numerous calls for guidance on how to use technology-based tools to enable effective and high-quality practices [27]. Technology-based tools pose new challenges to effective communication, with clinicians having to manage a new element of technological disruptions and operational talk (ie, guiding their patients’ participation in a technology-based encounter) alongside routine rapport building and clinical talk [28]. In addition to engaging in new forms of communication, clinicians have to adapt the old forms of communication to the technology-based environment. For example, certain clinical assessment skills do not readily translate to a technology-based context, such as the ability to assess patient knowledge about an examination. To address this, a combination of descriptive instructions and visual depictions has been offered as an adaptation to support clinical assessment in a technology-based environment [29]. Our findings add to this literature by focusing more specifically on providing actionable insights into *how* individual clinicians can enact the compassion element of quality throughout a technology-based encounter as well as how health systems can approach building compassionate competencies among the clinical workforce. These insights target the oft-cited barriers to understanding best practices and translating clinical and relational skills via a digital medium. Specifically, our results reinforce that training should highlight approaches for communicating compassionately via technology-based care as well as building clinical competency (ie, providing practical guidance on how to perform examinations and assessments over video) to increase clinician comfort and confidence [30,31]. Training interventions can leverage behavior change techniques, including demonstrating the behavior, providing instruction on how to perform the behavior, giving feedback on the behavior, and practicing the behavior [32]. The use of technology-based patient simulation provides a promising approach, supporting the development of specific verbal and nonverbal communication skills through practice while simultaneously building confidence [33].

### Aligning Patients and Clinicians to Establish Compassionate Hybrid Models

Patients and clinicians hold the shared belief that a hybrid model of in-person and technology-based care is the best. However, our results highlight a disconnect between patients’ preferences, needs, and capabilities and the ways in which hybrid care is currently being offered (or not) as well as received (or not). Although training is a well-suited strategy for eliciting preferences and building skills where there are deficits, it ignores the social drivers that lead to digital exclusion, thereby undermining compassion for those experiencing poverty, social exclusion, language, or literacy challenges [34].

Aligning the design and delivering of technology-based care to what is important and meaningful to both patients and providers is an essential element for establishing coherence between the 2 groups [35]. This includes establishing the meaningfulness (coherence) of the digital medium to individuals’ lives and work as a key driver of adoption and appraisal [35]. When video is determined to be an appropriate and mutually accepted modality, it is important to establish that the internet connection is adequate before initiating the formal aspects of the visit to ensure that the shared objectives are able to be met [29]. With sufficient technical connectivity, both primary care clinicians and patients reported an increased focus during technology-based consultations compared with telephone consultations. Of note, as with many of these findings, a preexisting relationship between the clinician and patient is an important moderating factor [36]. To further support meaningful interaction and address the persistent challenge of time constraints, training patients to effectively elicit information and actively engage in decision-making will promote the effective use of time, which is believed to be a more precise driver of patient outcomes than absolute time [37].

### Focusing on Enablement to Mitigate Burnout and Align Expectations

Health systems must also attend to the logistical strategies for integrating and routinizing technology-based alongside in-person care. It is important to recognize that these behaviors occur in the context of a broader clinical encounter and set of workflows brought about by the physical presence of the attending patient [28,34]. Just as physical practices were established and embedded based on time, physical spaces, and available materials [28], reimagining and reroutinizing the central functions of identifying, scheduling, rebooking, and monitoring patient appointments will require up-front efforts. Health care routines are often interdependent with other routines, highlighting that the risk to sustained implementation of video consultations lies not in the technology itself but in the considerable work involved in aligning administrative routines to accommodate clinical innovation [28].

Enablement strategies increase the means (or decrease the barriers) to improve the capability or opportunity to engage in compassionate behaviors [38]. Burnout is a consistent and major barrier in health care, which has been exacerbated by the shift in patient-clinician interactions, specifically, the depersonalization of care within a digital environment and the perceived decrease in value and respect for the clinician’s time. Addressing clinician burnout requires moving beyond educational strategies. To better support clinicians, the physical and social context of primary care needs to change to better align with individual as well as organizational values within the health care system [9]. Simply put, we need to change the clinical environment instead of trying to change the clinician. Interventions focusing on reducing workload, incorporating discussion meetings to enhance teamwork within interdisciplinary environments, and structural changes (eg, improving workflow) are considerably more likely to reduce burnout than interventions directly targeting clinicians to manage their own experiences of burnout [39].

Enablement strategies must also extend to patients and the public, as they are key players in health care interactions. Receiving gratitude from their patients regarding the quality of care mitigates burnout and motivates clinicians, underscoring the value of exploring how to amplify compassion satisfaction among patients and the public [40].

### Limitations

Although our results identified specific behaviors and the factors that drive their occurrence, we did not prospectively design this study as a comprehensive exploration of the behaviors that exist within a technology-based care interaction or the mechanisms through which they impact the experience of compassion. For example, although participants in our study described the importance of effective communication and the behaviors that enact it, we were unable to expand on *why* the identified behaviors made a difference to the experience of compassionate care. As a result, further direct exploration is needed to understand the totality of technology-based encounters, the behaviors that occur within them, the mechanisms through which they impact the experience of compassion, and their interaction with related routines and workflows. Our data reflect participant self-reports, and future work should focus on direct observations of patient and clinician behaviors to further elucidate and validate the nature and impact of technology-based interactions. Given the unanticipated breadth of insights into contextual drivers, there is a need to empirically understand how the existing policies and regulations in primary care impact the behaviors at an individual clinician level (vs understanding trends at a population level) to better inform health system transformations at a policy level. Given the restrictions of the COVID-19 pandemic at the time of this study, recruitment occurred via social media and digital distribution, which may have introduced bias into our sample. Specifically, as participants were recruited through social media platforms, we might anticipate that they could be more familiar with technologies as compared with those who do not use these platforms as part of their day-to-day lives. Currently, the relationship between an individual’s technology comfort and how they come to understand the elements of compassion remains unclear. Future work should explore whether and how the degree of prior experience and comfort with technology influences the perceptions of compassionate behaviors. Finally, our findings are not generalizable to the unique needs and experiences of excluded or underrepresented individuals or groups and the ways in which clinician behaviors do or should adapt to these unique needs and voices. Our sample is also skewed toward a younger demographic that is not representative of the demographic that accesses health care the most frequently. Understanding how the experiences of frequent users of the health care system contrast with these findings and the ways in which technology integration can create efficiencies for more compassionate in-person encounters is central to understanding how we move toward a more compassionate system for all.

### Conclusions

Despite the challenges introduced by the COVID-19 pandemic, our findings support the possibility of patient’s experiencing compassionate technology-based care. Although much of the patient experience is influenced by clinician behavior, clinicians need a supportive system and adequate resources to evolve new ways of working. The current state of technology-based care operationalization has led to widespread burnout, societal pressure, and shifting expectations of both clinicians and the health system more broadly, threatening the ability to deliver compassionate care. For clinicians to exhibit compassionate behaviors, they need more than just adequate supports; they also need to receive compassion from and experience the humanity of their patients. The high number of contextual drivers illustrates how the provision of compassionate care is closely embedded in a broader structure, organizations, and system that are beyond the control of clinicians, affecting their practice and behaviors. There is a need to create systemic compassionate conditions that enable clinicians to operate under the best circumstances to provide the expected quality of care, irrespective of the medium of care delivery.

## Appendix

Multimedia Appendix 1 Recruitment list.

Multimedia Appendix 2 Data collection tools.

Multimedia Appendix 3 Contextual attributes driving clinicians’ behaviors in technology-based interactions.

## Abbreviations

APQIP: Assessment Process for Quality Improvement Projects
COREQ: Consolidated Criteria for Reporting Qualitative Research
EMPaCT: Equity-Mobilizing Partnerships in Community

## Data Availability

An aggregate summary of the data generated during this study is presented in this published manuscript. Individual data transcripts cannot be publicly shared because of confidentiality.

## References

[1] Glazier RH, Green ME, Wu FC, Frymire E, Kopp A, Kiran T (2021). Shifts in office and virtual primary care during the early COVID-19 pandemic in Ontario, Canada. CMAJ.

[2] Bhatia RS, Chu C, Pang A, Tadrous M, Stamenova V, Cram P (2021). Virtual care use before and during the COVID-19 pandemic: a repeated cross-sectional study. CMAJ Open.

[3] Hutchings R (2020). The impact of COVID-19 on the use of digital technology in the NHS. Nuffield Trust.

[4] Gilbert AW, Billany JC, Adam R, Martin L, Tobin R, Bagdai S, Galvin N, Farr I, Allain A, Davies L, Bateson J (2020). Rapid implementation of virtual clinics due to COVID-19: report and early evaluation of a quality improvement initiative. BMJ Open Qual.

[5] Kilvert A, Wilmot EG, Davies M, Fox C (2020). Virtual consultations: are we missing anything?. Pract Diabetes.

[6] Hardcastle L, Ogbogu U (2020). Virtual care: enhancing access or harming care?. Healthc Manage Forum.

[7] Feo R, Kitson A, Conroy T (2018). How fundamental aspects of nursing care are defined in the literature: a scoping review. J Clin Nurs.

[8] Strauss C, Lever Taylor B, Gu J, Kuyken W, Baer R, Jones F, Cavanagh K (2016). What is compassion and how can we measure it? A review of definitions and measures. Clin Psychol Rev.

[9] Dewar B, Nolan M (2013). Caring about caring: developing a model to implement compassionate relationship centred care in an older people care setting. Int J Nurs Stud.

[10] Tehranineshat B, Rakhshan M, Torabizadeh C, Fararouei M (2019). Compassionate care in healthcare systems: a systematic review. J Natl Med Assoc.

[11] Uygur J, Brown JB, Herbert C (2019). Understanding compassion in family medicine: a qualitative study. Br J Gen Pract.

[12] Suggs LS (2006). A 10-year retrospective of research in new technologies for health communication. J Health Commun.

[13] Barak A, Grohol JM (2011). Current and future trends in internet-supported mental health interventions. J Technol Hum Serv.

[14] Wiljer D, Charow R, Costin H, Sequeira L, Anderson M, Strudwick G, Tripp T, Crawford A (2019). Defining compassion in the digital health age: protocol for a scoping review. BMJ Open.

[15] Baguley SI, Pavlova A, Consedine NS (2022). More than a feeling? What does compassion in healthcare 'look like' to patients?. Health Expect.

[16] Lawrence K, Hanley K, Adams J, Sartori DJ, Greene R, Zabar S (2020). Building telemedicine capacity for trainees during the novel coronavirus outbreak: a case study and lessons learned. J Gen Intern Med.

[17] Tong A, Sainsbury P, Craig J (2007). Consolidated criteria for reporting qualitative research (COREQ): a 32-item checklist for interviews and focus groups. Int J Qual Health Care.

[18] Martin D, Miller AP, Quesnel-Vallée A, Caron NR, Vissandjée B, Marchildon GP (2018). Canada's universal health-care system: achieving its potential. Lancet.

[19] Marchildon GP, Allin S, Merkur S (2020). Canada: health system review. Health Systems in Transition.

[20] Haggerty JL, Reid RJ, Freeman GK, Starfield BH, Adair CE, McKendry R (2003). Continuity of care: a multidisciplinary review. BMJ.

[21] Steele Gray C, Baker GR, Breton M, Kee K, Minkman M, Shaw J, Tietschert MV, Wankah P, Wodchis WP, Zonneveld N, Nies H, Waring J, Denis JL, Pedersen AR, Tenbensel T (2021). Will the “new” become the “normal”? Exploring sustainability of rapid health system transformations. Organising Care in a Time of COVID-19: Implications for Leadership, Governance and Policy.

[22] Saunders B, Sim J, Kingstone T, Baker S, Waterfield J, Bartlam B, Burroughs H, Jinks C (2018). Saturation in qualitative research: exploring its conceptualization and operationalization. Qual Quant.

[23] Braun V, Clarke V (2006). Using thematic analysis in psychology. Qual Res Psychol.

[24] Nowell LS, Norris JM, White DE, Moules NJ (2017). Thematic analysis: striving to meet the trustworthiness criteria. Int J Qual Methods.

[25] Squires JE, Aloisio LD, Grimshaw JM, Bashir K, Dorrance K, Coughlin M, Hutchinson AM, Francis J, Michie S, Sales A, Brehaut J, Curran J, Ivers N, Lavis J, Noseworthy T, Vine J, Hillmer M, Graham ID (2019). Attributes of context relevant to healthcare professionals' use of research evidence in clinical practice: a multi-study analysis. Implement Sci.

[26] (2019). Ontario expanding digital and virtual health care. Ontario Newsroom.

[27] de Vera K, Challa P, Liu RH, Fuller K, Feroz AS, Gamble A, Leung E, Seto E (2022). Virtual primary care implementation during COVID-19 in high-income countries: a scoping review. Telemed J E Health.

[28] Wherton J, Shaw S, Papoutsi C, Seuren L, Greenhalgh T (2020). Guidance on the introduction and use of video consultations during COVID-19: important lessons from qualitative research. BMJ Lead.

[29] Seuren LM, Wherton J, Greenhalgh T, Cameron D, A'Court C, Shaw SE (2020). Physical examinations via video for patients with heart failure: qualitative study using conversation analysis. J Med Internet Res.

[30] Brice S, Almond H (2020). Health professional digital capabilities frameworks: a scoping review. J Multidiscip Healthc.

[31] Nazeha N, Pavagadhi D, Kyaw BM, Car J, Jimenez G, Tudor Car L (2020). A digitally competent health workforce: scoping review of educational frameworks. J Med Internet Res.

[32] Michie S, Richardson M, Johnston M, Abraham C, Francis J, Hardeman W, Eccles MP, Cane J, Wood CE (2013). The behavior change technique taxonomy (v1) of 93 hierarchically clustered techniques: building an international consensus for the reporting of behavior change interventions. Ann Behav Med.

[33] Peddle M, Bearman M, Nestel D (2016). Virtual patients and nontechnical skills in undergraduate health professional education: an integrative review. Clin Simul Nurs.

[34] Greenhalgh T, Shaw S, Wherton J, Vijayaraghavan S, Morris J, Bhattacharya S, Hanson P, Campbell-Richards D, Ramoutar S, Collard A, Hodkinson I (2018). Real-world implementation of video outpatient consultations at macro, meso, and micro levels: mixed-method study. J Med Internet Res.

[35] Steele Gray C, Gravesande J, Hans PK, Nie JX, Sharpe S, Loganathan M, Lyons R, Cott C (2019). Using exploratory trials to identify relevant contexts and mechanisms in complex electronic health interventions: evaluating the electronic patient-reported outcome tool. JMIR Form Res.

[36] Donaghy E, Atherton H, Hammersley V, McNeilly H, Bikker A, Robbins L, Campbell J, McKinstry B (2019). Acceptability, benefits, and challenges of video consulting: a qualitative study in primary care. Br J Gen Pract.

[37] Dugdale DC, Epstein R, Pantilat SZ (1999). Time and the patient-physician relationship. J Gen Intern Med.

[38] Michie S, van Stralen MM, West R (2011). The behaviour change wheel: a new method for characterising and designing behaviour change interventions. Implement Sci.

[39] Linzer M, Poplau S, Grossman E, Varkey A, Yale S, Williams E, Hicks L, Brown RL, Wallock J, Kohnhorst D, Barbouche M (2015). A cluster randomized trial of interventions to improve work conditions and clinician burnout in primary care: results from the healthy work place (HWP) study. J Gen Intern Med.

[40] Sacco TL, Copel LC (2018). Compassion satisfaction: a concept analysis in nursing. Nurs Forum.

