# Supplementary material for: Building Compassionate Experience Through Compassionate Action: Qualitative Behavioral Analysis
Source: JMIR Form Res. 2023 May 31;7:e43981. doi: 10.2196/43981 (PMC10267792; doi:10.2196/43981)
Supplement: Multimedia Appendix 2 [file formative_v7i1e43981_app2.docx]

## Multimedia Appendix

**Interview guide for patients who have engaged with virtual care**

Before we begin the interview, can you please let me know whether you’ve had the chance to complete the short demographic questions sent in the online survey? (If no, will send reminder email after interview)

| **Topics** | **Questions** | **Probes** |
| --- | --- | --- |
| Introduction | Why are you interested in participating in this interview/study today? |  |
| Context setting | Before we begin the interview, we want to acknowledge how personal background and lived experiences can impact an individual’s experience with the health care system and the care they receive from their doctor or nurse. We refer to this as context. Context can be the things that patients think their healthcare providers need to be aware of. We’ll reflect on this context at the end as well but feel free to draw on these points when it makes sense for you. Please know that any topic is welcome for discussion. |  |
| Perceptions of compassionate care | For the purpose of this study, we define compassionate care as:   - Awareness of another’s experience or need - Appraising one’s own role and abilities in an interaction - Being aware of context   but we want to hear in your own words, what does compassionate care mean to you? | How do you know you’re receiving compassionate care? /what does that *feel* like to you? |
|  | To what degree has your family doctor/nurse expressed awareness of another’s (your) experience or need?    Can you walk me through what the interaction was like? | What made you feel that compassionate care was present? |
|  | To what degree has your family doctor/nurse considered their own role (responsibilities) and abilities in an interaction (with you)?  Can you walk me through what the interaction was like? | What made you feel that compassionate care was present |
|  | To what degree has your family doctor/nurse been aware of (your/their own) context (other factors/circumstances that influence your health/life)?  Can you walk me through what the interaction was like? | What made you feel that compassionate care was present |
|  | What aspects of health matter to you? |  |
|  | Primary care is unique in that you have the opportunity to interact with your family doctor/or nurse over time, how long have you known your family doctor/nurse? Can you describe the nature of your relationship with them? Do you have a strong relationship with them? | **Does** that impact your experience of compassionate care?  **Do** you think not having a strong relationship would impact your experience of compassionate care?  How did you build that relationship with your family doctor/nurse?  If relationship is not strong: What would make the relationship better? |
|  | Please complete this sentence for me:  1. As a patient my responsibility is: ________________.  2. My physicians responsibility is: _________________. | How does your perception/experiences of compassionate care align with your perception of the responsibility of your family doctor/nurse? |
| Experiences of digital care | We define digital care as any healthcare interaction that uses technology (ex. Emailing, video visits, remote monitoring, etc.). Is this definition clear? In what other ways have you received digital care with your family doctor and nurse? |  |
|  | Can you describe your experiences using technology with your family doctor/nurse? | Can you walk me through a typical virtual care visit?  What was the reason for your consultation?    What visit options were available to you and how were they presented?    What did you like, what did you not like?    How does this compare to an in-person visit? |
| Intersection of compassionate care and digital care | Within your digital care interactions, how has your family doctor or nurse shown you compassionate care?  What did your family doctor or nurse do that made you feel that it was compassionate? | Is there something they could/should have done? what was most effective? Why was it most effective? (really trying to understand on what it meant to the participant)    Has using technology influenced your experiences of receiving compassionate care? |
|  | What has your experience been like using technology to access your healthcare? | How similar is that experience with technology outside of healthcare?    Is there anything else that could be done to make you feel more comfortable with technology?    Do you have what you need in order to access care digitally? |
|  | Have you ever been asked about your experiences using technology to access care? | Has your family doctor/nurse ever asked you? |
|  | In what instances do you prefer to meet your family doctor/nurse in person versus virtually? | And how does that link back to having a compassionate interaction? |
|  | How would you describe your ideal form of virtual care? What does that look like to you? |  |
| Closing and context setting | Keeping in mind the role of context in compassionate care, how do you feel your personal background and lived experiences influences your experience of receiving compassionate care digitally? We invite you to reflect on how your race, gender and other sociodemographic factors including language, and disability may impact both the care you receive and your experiences within the healthcare system. | How does your family doctor or nurse’s background (race, linguistic, gender etc.) influence your comfort level? |
|  | Is there anything else you want to share with me about compassionate care and digital health? |  |

Those are all the questions I had. Is there anything else you would like to add on this topic that we have not already covered?

Thank you for participating.

STOP RECORDING NOW

**Interview guide for patients who have not engaged with virtual care**

Before we begin the interview, can you please let me know whether you’ve had the chance to complete the short demographic questions sent in the online survey? (If no, will send reminder email after interview)

1. experiences of health care 2. What does your ideal form of health care look like to you? 3. What does compassionate care mean to you? 4. is there a role for digital health to support delivering the key components of what compassionate care is to you?

| Topics | Questions | Probes |
| --- | --- | --- |
| Introduction | Why are you interested in participating in this interview/study today? |  |
| Context setting | Before we begin the interview, we want to acknowledge how personal background and lived experiences can impact an individual’s experience with the health care system and the care they receive from their doctor or nurse. We refer to this as context. Context can be the things that patients think their healthcare providers need to be aware of. We’ll reflect on this context at the end as well but feel free to draw on these points when it makes sense for you. Please know that any topic is welcome for discussion. |  |
| Perceptions of compassionate care | For the purpose of this study, we define compassionate care as:  Awareness of another’s experience or need  Appraising one’s own role and abilities in an interaction  Being aware of context  but we want to hear in your own words, what does compassionate care mean to you? | How do you know you’re receiving compassionate care? /what does that feel like to you ? |
|  | To what degree has your family doctor/nurse expressed awareness of another’s (your) experience or need?    Can you walk me through what the interaction was like? | What made you feel that compassionate care was present? |
|  | To what degree has your family doctor/nurse considered their own role (responsibilities) and abilities in an interaction (with you)?  Can you walk me through what the interaction was like? | What made you feel that compassionate care was present |
|  | Primary care is unique in that you have the opportunity to interact with your family doctor/or nurse over time, how long have you known your family doctor/nurse? Can you describe the nature of your relationship with them? Do you have a strong relationship with them? | **Does** that impact your experience of compassionate care?  **Do** you think not having a strong relationship would impact your experience of compassionate care? |
|  | What aspects of health matter to you? |  |
|  | Please complete this sentence for me:  1. As a patient my responsibility is: ________________.  2. My physicians responsibility is: _________________. | How does your perception/experiences of compassionate care align with your perception of the responsibility of your family doctor/nurse? |
| **DIGITAL DIVIDE**  (trying to understand which level of the digital divide applies: access, capability, outcome) | How comfortable do you feel using technology? Would you feel adequately prepared (i.e. technical skill, access) to use technology when accessing care? | Is there anything else that could be done to make you feel more comfortable with technology? |
|  | What resources and conditions do you need to meaningfully engage in digital health? *Provide examples if needed i.e.,* video conferencing, high-speed internet | Do you have what you need to access care digitally? |
|  | Has your family doctor/nurse ever offered you digital health care visits? | How could using technology with your family doctor/nurse change your experience of receiving healthcare? |
| **MISTRUST/FEAR/STIGMA**  (trying to understand how their previous experiences affects their concerns about digital health) | Is there a reason as to why you have not engaged in digital healthcare so far? If there is, can you please share why that is? | What concerns do you have about using digital health? |
|  | How do you think your past experiences with compassionate care and your family doctor/nurse affect your willingness to use digital health technology? |  |
|  | In what instances do you think you would prefer to meet your family doctor/nurse in person versus virtually? | And how does that link back to having a compassionate interaction? |
| **CULTURAL COMPETENCE**  (how to tailor the digital health experience to the patient and ensure it is meaningful) | Under what circumstances would you be willing to use digital health? | What do you think needs to be done to make you feel comfortable enough to meaningfully engage in digital care? |
|  | How would you describe your ideal form of virtual care? What does that look like to you? | What format of digital health would be the easiest/most convenient for you to use? |
|  | Keeping in mind the role of context in compassionate care, how do you feel your personal background and lived experiences influences your experience of receiving compassionate care? We invite you to reflect on how your race, gender and other sociodemographic factors including language, and disability may impact both the care that you receive and your experiences within the healthcare system |  |
|  | How do you think your family doctor/nurse’s identity influences your experience with the healthcare system? | How does your family doctor or nurse’s background (race, linguistic, gender etc.) influence your comfort level?  What can your provider do better to provide you care that considers your context? |
| **COMMUNITY SUPPORT**  (what additional support is needed and if patients are open to it) | What support do you need to ensure you can engage in digital health care? |  |
| Closing | Is there anything else you want to share with me about compassionate care and digital health? |  |

Those are all the questions I had. Is there anything else you would like to add on this topic that we have not already covered?

Thank you for participating.

STOP RECORDING NOW

**Interview guide for primary care nurses and physicians**

Before we begin the interview, can you please let me know whether you’ve had the chance to complete the short demographic questions sent in the online survey? (If no, will send reminder email after interview)

Preamble for nurses only: Who am I? My background as a nurse; I spent the most part of my practice in doing research; in using asynchronous education program to share knowledge with patients having chronic care conditions.

English is not my first language. I will reflect you my understanding of your thoughts and experience; and perhaps ask to summarize or clarify. This is to ensure that I have an accurate understanding of your experience. And please, don’t hesitate to let me know if some questions need clarification.

Two of my colleagues are also in the Zoom, for note taking and observing the flow of the interview. Are you comfortable with that?

In this interview, we will cover these topics: context setting and how your background can influence the care you provide to patient; your perceptions of the meaning of compassionate care, your experiences in providing digital care; and the intersection of compassionate care and digital care.

| **Topics** | **Questions** | **Probes** |
| --- | --- | --- |
| **Introduction** | Why are you interested in participating in this interview/study today? |  |
| **Context setting** | Before we begin the interview, we want to acknowledge how context can impact an individual’s experience with the health care system and the care they receive or provide. We invite you to reflect on how your race, gender and other sociodemographic factors including language, and disability may impact both the care that you provide and your experiences within the healthcare system. We’ll reflect on this context at the end as well but feel free to draw on these points when it makes sense for you. |  |
| **Perceptions of compassionate care** | This is the definition of compassionate care, but we want to hear in your own words, what does compassionate care mean to you?  For the purpose of this interview, compassionate care is described as:  Awareness of another’s experience or need  Appraising one’s own role and abilities in an interaction  Being aware of context | How do you know you’re providing compassionate care? /what does that feel like to you |
|  | To what degree have you had that kind of compassionate care interaction with your patients? Can you walk me through what the interaction was like? | What made you feel that compassionate care was present? |
|  | How does your perception/experiences of compassionate care align with your perception of your role as a family doctor/nurse? |  |
|  | Can you walk me through a situation where you had an in-person appointment with a patient and highlight instances where you felt you showed compassion in the interaction? | How do you know you are providing compassionate care? |
| **Experiences of digital care** | We define digital care as any healthcare interaction that uses technology (ex. Emailing, video visits, remote monitoring, etc). Does this definition of digital health resonate with you? If not, how would you describe digital health? |  |
|  | Can you describe some of your key experiences of using digital health with your patients? | Can you walk me through a typical virtual care visit?  What visit options were available to your patient and how were they presented?  How does this compare to your experiences providing in-person care? |
|  | What resources and conditions (i.e. video conferencing, training workshops, high-speed internet) are needed for you to meaningfully engage in a digital interaction? |  |
|  | What skills would you need to meaningfully engage in compassionate care with your patients when connecting via digital technologies? | What skills do you think your patients need to meaningfully engage in a digital interaction? |
| **Intersection of compassionate care and digital care** | How does technology influence how you provide care? | Does it make it easier or more difficult in your role as a family doctor/nurse?  Are there tasks or activities (i.e. building rapport, scheduling appointments) that look different in a digital interaction compared to in-person care? |
|  | How do you generate a relational connection with patents in a digital interaction? | How do you think these strategies translated for your patients?  Have you been provided feedback from your patients about their experiences with using digital health? |
|  | Reflecting on the discussion we had earlier on the definition of compassion. How does the compassionate care you provide show up in a digital interaction with your patient? | How does this compare to the compassionate care that you are able to provide when giving in-person care?  How do you know that the care you provide to your patients is perceived as compassionate? |
|  | In what instances do you prefer to meet your patient in-person versus virtually? | And how does that link back to having a compassionate interaction? |
|  | What form of digital health do you think is the most effective at providing compassionate care to your patients? |  |
|  | What do you think influences your patients’ experience when using technology in their digital interaction (i.e., language, geographic, race, etc.)? | To what degree does your patient’s comfort with technology (i.e., technical skill, access) influence their experience of care? |
| **Closing and context setting** | Keeping in mind the role of context in compassionate care, how do you feel your personal background and lived experiences influences the compassionate care that you provide digitally? | How does your identity influence your patients’ experience? |
|  | Is there anything else you want to share with me about compassionate care and digital health? |  |

Those are all the questions I had. Is there anything else you would like to add on this topic that we have not already covered?

Thank you for participating.

STOP RECORDING NOW
