# Supplementary material for: Building Compassionate Experience Through Compassionate Action: Qualitative Behavioral Analysis
Source: JMIR Form Res. 2023 May 31;7:e43981. doi: 10.2196/43981 (PMC10267792; doi:10.2196/43981)
Supplement: Multimedia Appendix 3 [file formative_v7i1e43981_app3.docx]

## Multimedia Appendix

**Table S1. Contextual attributes driving clinicians’ behaviours in virtual interactions**

| Attribute | Description | Supporting Quote |
| --- | --- | --- |
| Resource Access | Access to technology and a lack of centralized support to learn a new way of working. | *“I think maybe troubleshooting skills because a lot of the time the person on the other end sometimes they would get error messages or like they couldn’t join the virtual visit with their doctor and they’d end up calling the clinic asking for tech support and, yeah, there was little to no capacity to provide tech support to people. So if it didn’t work the first time, like if the virtual just didn’t work, it would end up being a telephone. So maybe some tech support.” Registered Nurse 2, Female* |
| Funding structures | Funding system and billing structures influence how care is delivered and experienced via clinician behaviour, nature of visit, length of consultation, and virtual logistics. | *“And I have the luxury of time as a nurse practitioner, right. I'm not fee for service, I'm salary, so if I see twenty patients in a clinic or five, I can say the exact same. So that’s really nice for me, so I can allocate the appropriate amount of time for the patient, so balancing access and need, and knowing that some patients just need more time. But I'm not mandated to see all of these people, because I have a roster of two thousand patients who all need help.” Nurse Practitioner 5, Female*  “*I think that we have to really start looking at health care as an essential service, but as a compassionate people service not an efficient, cost-cutting machine which I think it is right now. It’s just a machine. I look at hospitals as like a garage.”* Patient 22, Female |
| Culture | Building relational connections in person is central to primary care however there is a need to learn how to build these connections via digital technologies. | *“It’s the loss of the in-person energy and all the information that comes from that in person encounter that enriches the encounter. It’s the just the lack of availability of patients or someone called it lack of respect that’s just not there. They don’t answer at the time of their appointment. So, we have to rebook and then there’s the whole question of you know, how do you treat people who are repeat offenders who don’t show up for their virtual visits. So, it’s just added on layers of administrative and experiential burden. It’s a burden.” Physician 13, Female*  *“If you took a transcript of our messaging and you made us say it an in-person interaction, I could say the exact same thing as I would and we can have the exact same interaction as we would on MyChart, but there could be non-verbal like a tone that I’m using in my voice or body language or something that still comes across as like more genuine, more authentic, more empathetic than it would over digital, right. Like literally if you took the exact same words but put us in an in-person, I wouldn’t say it’s the exact same interaction.” Registered Nurse 1, Female* |
| Regulatory Standards | Lack of evidence-based standards and best-practice guidance leads to a lack of standardization. | *“If they reach out to me virtually and I say, given what you’re telling me, I really need to see you in person, because there’s a physical assessment component here. Most of them have been good, some of them have been like, “No, just order testing.” and I’m like, “That’s not, I still have a standard of practice that I have to maintain.” Nurse Practitioner 18, Female* |
| Societal influences | Expectation that health care delivery embraces and pushes virtual care and patient attitudes towards enhancing convenience. | *“I think patients who are demanding increased convenience without understanding that they're sacrificing quality of care or that they're OK with that, are dictating the way that medicine is going to go in the future for telemedicine. I don't think the pushback from physicians has been strong enough because they are – I mean we are all taking part of it in these telemedicine clinics. We are all dropping our level of care required because we're kind of accepting that this is how things are going to go.” Physician 10, Male* |
| Work Structure | Management of workloads, and the arrangement of workflows, schedules, and time to perform the tasks. | *“If I sit there for the first 15 minutes of my visit trying to figure out how to work technology and I’ve got 10 patients waiting out in the waiting room that I need to see, I’m not going to be able to be very compassionate, I’m not going to have the time to ask these kinds of questions, I’m not going to have the time to form that relationship. I’m going to be irritated that I’m behind, I’m going to be – my mind’s going to be in different places because I know that I’ve got patients waiting and now I’m behind, and I’m going to be frustrated because I can’t work this thing that should be pretty simple to work.” Nurse Practitioner 7, Female* |
| Patient characteristics and needs | Previous experience with technology, digital literacy, access to technology and private space, and stable internet connectivity. | “*They have to click at the right time because otherwise they won't connect. And it's just a disaster and doesn't work. I had a couple who I needed to see on [Ontario Telemedicine Network], and the patient and her husband were both trying – her husband works in IT and it took us half an hour to get connected. Like, that should not happen. So that resource – if we have a working platform that is easy to use would increase uptake by other clinicians and by patients.****”*** *Physician 11, Male*  *“My mom has come a long way in knowing how to use Zoom and having a webcam and having a computer and all those things that are potential barriers to digital health or digital anything else and still you often need a person with knowledge and expertise to be a navigator and a support, even when you have the infrastructure.*” *Patient 5, Female* |
